# PIWI-interacting RNA 57125 restrains clear cell renal cell carcinoma metastasis by downregulating CCL3 expression

**DOI:** 10.1038/s41420-021-00725-4

**Published:** 2021-11-03

**Authors:** Lifeng Ding, Ruyue Wang, Wanjiang Xu, Danyang Shen, Sheng Cheng, Huan Wang, Zeyi Lu, Qiming Zheng, Liya Wang, Liqun Xia, Gonghui Li

**Affiliations:** Department of Urology, Sir Run Run Shaw Hospital, Zhejiang University School of Medicine, Hangzhou, China

**Keywords:** Renal cell carcinoma, Cell invasion

## Abstract

Clear-cell renal cell carcinoma is one of the most common tumors disagnosed, with nearly one third of patients diagnosed with metastatic ccRCC. Although an increasing number of studies has revealed that piwi-interacting RNAs are aberrantly expressed in diverse types of cancers, few of them explored the detailed molecular mechanism of piRNAs in carcinogenesis, particularly in ccRCC. In this study, differentially expressed piRNAs associated with ccRCC were selected by using piRNA-sequencing combined with TCGA data analysis, and piR-57125 was identified. PiR-57125 was found remarkably downregulated in ccRCC samples. Functionally, knockdown of piR-57125 promoted migration and invasion of ccRCC, while overexpression of piR-57125 suppressed ccRCC metastasis. In vivo lung metastasis model also confirmed the same results. CCL3 was identified as the direct target of piR-57125 which could potentially reverse the inhibition effect of piR-57125 in ccRCC metastasis. Further study revealed that piR-57125 modulated ccRCC metastasis through the AKT/ERK pathway. These data indicate that piR-57125 restrains ccRCC metastasis by directly targeting CCL3 and inhibiting the AKT/ERK pathway, and could be a potential therapeutic target for ccRCC.

## Introduction

Clear-cell renal cell carcinoma (ccRCC) is the sixth most frequently diagnosed cancer around the world [1]. More than 17500 RCC patients died annually, accounting for nearly 2.5% of cancer deaths worldwide [2–4]. Because of the lack of specific symptoms, combined with high metastasis and recurrence rates, nearly one-third of renal cell carcinoma patients are diagnosed with metastatic ccRCC (mccRCC) [5, 6]. Meanwhile, ccRCC is highly resistant to chemotherapy and radiotherapy. Although there has been a considerable amount of research focused on RCC in recent decades, the detailed molecular mechanisms of RCC are still poorly understood [7]. Thus, a better understanding of the molecular mechanisms of metastatic RCC is critical for the diagnosis and treatment of mccRCC.

piRNAs are a subtype of small noncoding RNAs whose length are about 24-31 nucleotides, and which possess 2’-O- methylation at the 3’ end [8, 9]. piRNAs are derived from the single strand of precursor transcripts, unlike other small noncoding RNAs like miRNAs [9]. Generally, piRNAs accomplish their function by interacting with PIWI protein to form a piRNA-induced silencing complex (piRISC) [10, 11]. piRNAs are initially found in germline cells. However, with the development of high-throughput sequencing, emerging evidences have revealed that piRNAs also existed in somatic cells [12, 13]. Although little is known about piRNAs in cancer, a growing number of studies have revealed that specific piRNAs showed aberrant expression during tumorigenesis, and have the potential as predictive, diagnostic, or prognosis biomarkers [14–17]. However, few studies focus on the function and specific regulation mechanisms of piRNAs in carcinogenesis. Thus, it is meaningful to explore the ccRCC-related piRNAs and elucidate their specific mechanisms [18, 19].

In this current study, we found that piR-57125 was downregulated in the ccRCC tissues compared with adjacent normal tissues. Loss- and gain-of-function assays showed that piR-57125 act as a tumor suppressor gene that played an important role in ccRCC metastasis. Mechanism studies showed that piR-57125 directly bound to 3’UTR region of CCL3 mRNA, thereby inhibiting the downstream AKT/ERK pathway. Overall, our results showed that piR-57125 was a metastasis-related piRNA which could be a potential therapy target for metastatic ccRCC.

## Results

### piR-57125 is downregulated in ccRCC

**Fig. 1:**
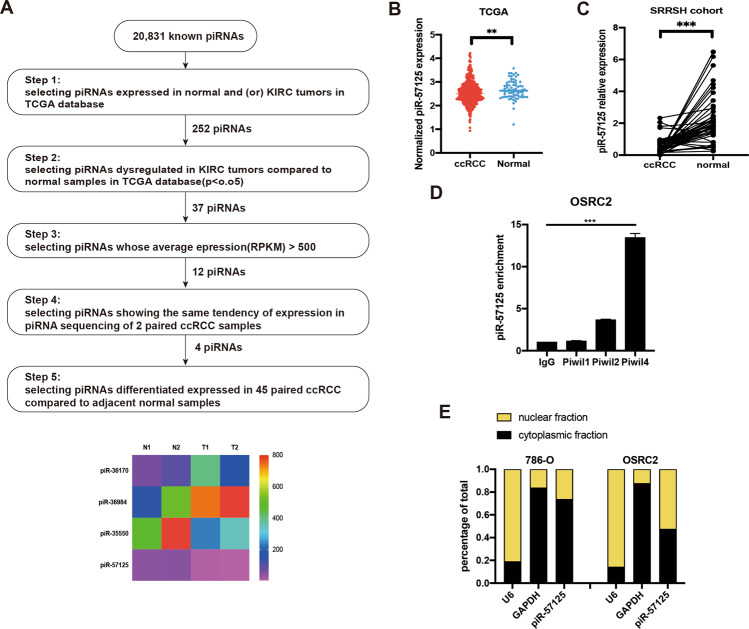
piR-57125 is downregulated in ccRCC. **a** Top, flow chart demonstrates the selection criteria of potential piRNAs dysregulated in ccRCC. Bottom, heatmap showing the differential expression of piRNAs in 2 pairs of ccRCC and adjacent normal samples by piRNA sequencing. **b** Expression of piR-57125 in ccRCC and normal patients based on the analysis of TCGA data. **c** qRT-PCR analysis of piR-57125 expression in 45 paired ccRCC and adjacent normal samples from patients recruited from the SRRSH cohort. Data represent mean ± S.D. **d** RIP and RT-qPCR assay showed that PIWIL4 has more affinity to piR-57125 in OSRC-2 cells compared with PIWIL1 and PIWIL2. **e** Cytoplasm and nuclear mRNA fractionation experiment showed that piR-57125 exists in both the cytoplasm and the nucleus. U6 and GAPDH act as the positive controls of nucleus and cytoplasm, respectively. **p < 0.01; ***p < 0.001.

To identify clear-cell renal cell carcinoma-associated PIWI-interacting RNA that could be involved in tumorigenesis, we firstly analyzed the expression profiles of piRNAs from The Cancer Genome Atlas (TCGA), which contains 529 ccRCC tissues and 71 adjacent normal tissues, which was characterized by Martinez, et al. [20]. Among all the piRNAs expressed in normal and (or) KIRC patients, we identified 12 piRNAs that met the following criteria: (1) dysregulated in KIRC tumors compared to normal samples; and (2) an average expression (RPKM) > 500. Next, we performed piRNA sequencing using 2 paired clear-cell carcinoma and matched adjacent non-tumor samples (GSE183496, Supplementary Table S1). Of the nine of the piRNAs, four were selected as the candidates for further validation (Fig. 1a). Finally, we selected piR-57125 as the candidate piRNA which may potentially contribute to ccRCC progression. We found that the expression of piR-57125 was downregulated in ccRCC tissues compared to the normal tissue in the TCGA database (Fig. 1b). This result was validated by another cohort which contained 45 paired ccRCC and adjacent normal tissues from Zhejiang University School of Medicine Sir Run Run Hospital (Fig. 1c). Previous studies have shown that piRNAs can directly bind to PIWIL protein to exert their function. By performing RNA immunoprecipitation followed by qRT-PCR, we found that piR-57125 had more affinity to PIWIL4 than to PIWIL1 or to PIWIL2 (Fig. 1d). The cellular distribution analysis showed that piR-57125 existed both in the cytoplasm and the nucleus (Fig. 1e), indicating that piR-57125 may regulate downstream gene expression at the post-transcriptional level.

### piR-57125 modulates migration and invasion of ccRCC cells

**Fig. 2:**
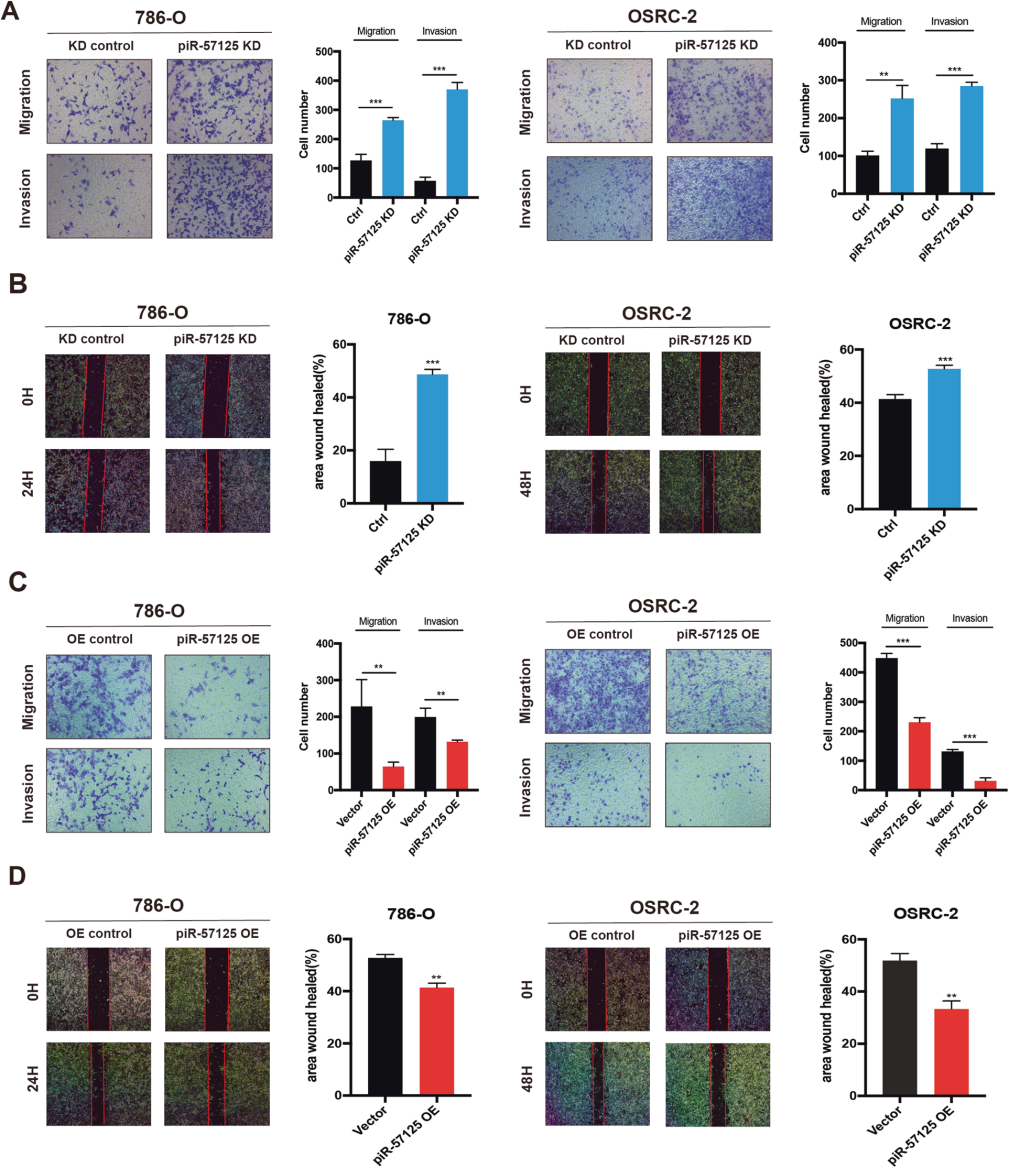
piR-57125 inhibits ccRCC metastasis. **a** Transwell assay showed that knockdown of piR-57125 promoted 786-O and OSRC-2 cell metastasis. Data represent mean ± S.D. from three independent experiments. **b** Wound healing assay demonstrated that knockdown of piR-57125 enhance 786-O and OSRC-2 cells’ metastatic ability. Data represent mean ± S.D. from three independent experiments. **c** Transwell assay showed that the metastatic ability of ccRCC cells was inhibited when piR-57125 was overexpressed. Data represent mean ± S.D. from three independent experiments. **d** wound healing assay revealed that overexpression of piR-57125 slowed the metastatic rate of 786-O and OSRC-2 cells. Data represent mean ± S.D. from three independent experiments. *p < 0.05; **p < 0.01; ***p < 0.001.

Since piR-57125 is downregulated in ccRCC patients, we asked whether piR-57125 functions as a tumor suppressor in tumorigenesis. Firstly, we examined the expression of piR-57125 in ccRCC cell lines. The results showed that piR-57125 was abundantly expressed in 786-O and OSRC-2 cells, compared with endogenous control (Supplementary Fig. 1a). We used a CCK-8 assay to determine the proliferation rate of ccRCC cells transfected with piR-57125 mimics or inhibitors, and the overexpression efficiency is evaluated by qRT-PCR (Supplementary Fig. 1b). Our results showed that neither overexpression nor knockdown of piR-57125 had much effect on ccRCC cells proliferation (Supplementary Fig. 2a and b). However, the transwell assay and wound healing assay showed that knockdown of piR-57125 significantly enhanced 786-O and OSRC-2 migration and invasion compared to the control treatment (Fig. 2a and b). Meanwhile, overexpression of piR-57125 inhibited the same cell lines’ migration and invasion (Fig. 2c and d).

### piR-57125 inhibits CCL3 expression by directly interact with CCL3 mRNA

**Fig. 3:**
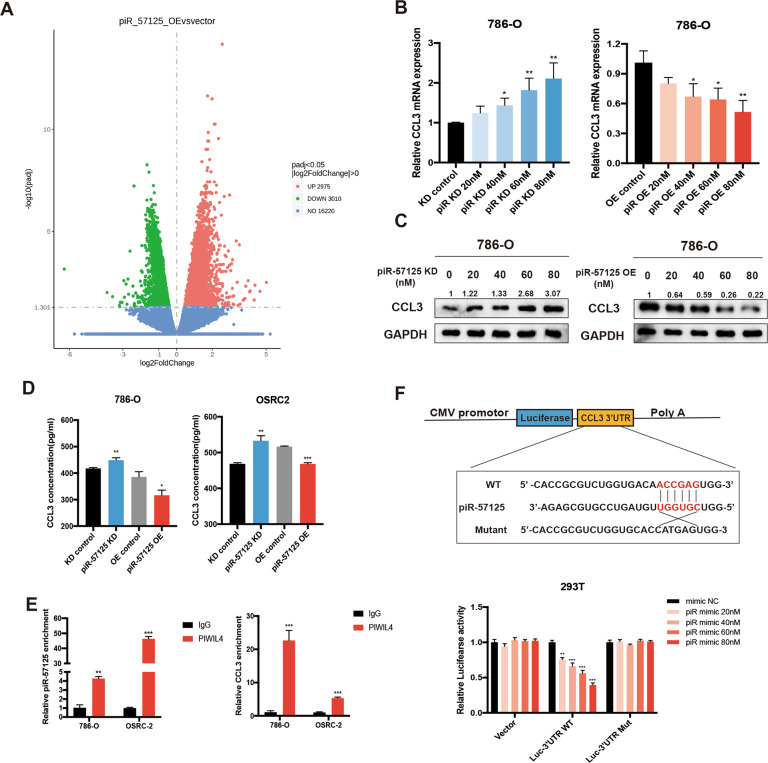
piR-57125 regulates CCL3 expression. **a** Volcano map represents the differentially expressed genes upon piR-57125 overexpression in OSRC-2 cells from RNA-seq. The experiments were carried out three times with the *p* < 0.05, |log2FC| > 0. **b**, **c** Decreased or increased mRNA (**b**) or protein (**c**) expression of CCL3 in 786-O cells via transfection with piR-57125 inhibitors or mimics respectively. Data represent mean ± S.D. from three independent experiments. **d** ELISA experiment showed that the secretion of CCL3 proteins from ccRCC cells was respectively increased or decreased upon knockdown or overexpression of piR-57125. Data represent mean ± S.D. from three independent experiments. **e** RIP and RT-qPCR assays revealed that piR-57125 and CCL3 mRNA had more affinity with PIWIL4 compared with the IgG control. Data represent mean ± S.D. from three independent experiments. **f** Luciferase reported plasmids which contained WT or Mut 3’UTR region of CCL3 were combined with piR-57125 or control mimics, and transfected into HEK-293T cells. The relative luciferase activity was normalized with renilla luciferase. Data represent mean ± S.D. from three independent experiments. *p < 0.05; **p < 0.01; ***p < 0.001.

To elucidate the specific mechanism by which piR-57125 restrains the ccRCC metastasis. Noncoding RNAs have been reported to have the cis regulation effect on the neighboring extrachromosomal genes [21]. Therefore, we firstly examined whether piR-57125 had this regulation effect on 8 genes (TTC37, POU5F2, SLF1, FAM81B, KIAA0825, FAM172A, MCTP1, NR2F1) within about 2 mega-bases centered on the piR-57125 gene locus. However, the results showed that overexpression or knockdown of piR-57125 did not affect the expression of these genes (Supplementary Fig. 3a), suggesting that piR-57125 may not function in this way. Previous studies found that piRNAs could interfere with the expression of DNMTs to regulate DNA methylation [22, 23]. However, we found that overexpression or knockdown of piR-57125 did not alter the mRNA and protein levels of DNMTs (Supplementary Fig. 3b and c). Next, we treated OSRC-2 with or without piR-57125 mimics and subsequently performed RNA sequencing. The results showed that there was a total of 2975 up-regulated and 3010 down-regulated mRNAs with a |fold change| > 0 and a corresponding *P* < 0.05, compared with the control treatment (Fig. 3a). Among these differentially expressed genes, we identified CCL3 as the candidate target gene of piR-57125 by verifying the result of RNA sequencing (Supplementary Fig. 4a). Then, we found that piR-57125 overexpression downregulated CCL3 expression while knockdown of piR-57125 upregulated it (Fig. 3b and Supplementary Fig. 5a). Furthermore, western blot results also confirmed that piR-57125 negatively regulated CCL3 expression in proteins level (Fig. 3c and Supplementary Fig. 5b). Meanwhile, piR-57125 overexpression also decreased the secretion of CCL3 and knockdown of piR-57125 enhanced it, as determined by ELISA assay (Fig. 3d).

Previous studies have showed that piRNAs could form a RNA-induced silencing complex (RISC) by interacting with PIWIL protein to regulate mRNA expression. So, we performed RNA immunoprecipitation followed by qRT-PCR, showing that piR-57125 and CCL3 had more affinity to PIWIL4 compared with the IgG control group (Fig. 3e). These findings indicated that piR-57125 may decrease CCL3 expression in a PIWIL4-dependent RISC mechanism. To verify this hypothesis, we performed target site prediction by using RNAhybrid (https://bibiserv.cebitec.uni-bielefeld.de/) [24]. We then performed dual luciferase reporter gene assays to confirmed the prediction. HEK-293T cells were transfected with luciferase plasmids harboring the WT or MUT 3’UTR sequence of CCL3 combined with piR-57125 or scramble mimics, and the luciferase activity was measured after 48 h. We found that piR-57125 mimics could significantly decrease the luciferase activity compared with scramble mimics. However, we did not observe any change in luciferase activity in MUT plasmids co-transfected with piR-57125 or scramble mimics (Fig. 3f). These results led us to the conclusion that piR-57125 decreases the expression of CCL3 by directly binding to the 3’UTR of CCL3.

### CCL3 expression is upregulated in ccRCC and promotes ccRCC cells metastasis

**Fig. 4:**
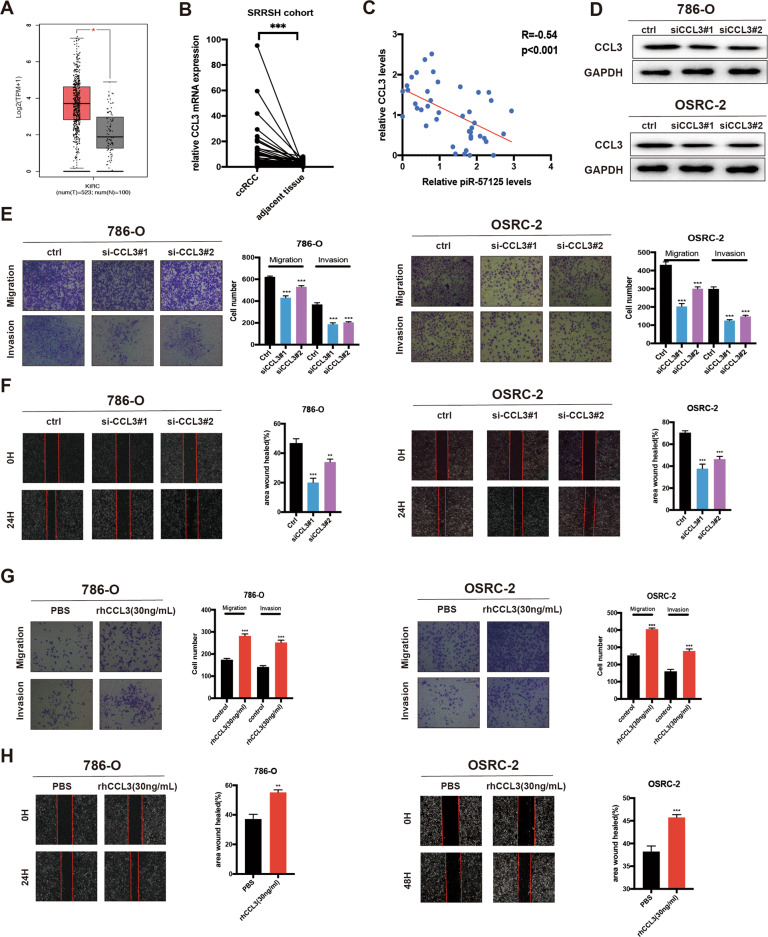
CCL3 is upregulated in ccRCC and promotes ccRCC metastasis. **a** Expression of CCL3 in ccRCC and normal patients based on analysis of TCGA data. **b** RT-qPCR analysis of CCL3 expression in 45 paired ccRCC and adjacent normal samples from patients recruited from the SRRSH cohort. **c** correlation analysis showed that piR-57125 was negatively correlated with CCL3 expression in ccRCC patients from the SRRSH cohort. **d** Western blot analyzed the knockdown efficiency of CCL3 siRNAs. **e** Transwell assay revealed that knockdown of CCL3 restrained 786-O and OSRC-2 cells metastasis. Data represent mean ± S.D. from three independent experiments. **f** Wound healing assay demonstrated that knockdown of CCL3 inhibited 786-O and OSRC-2 cells metastatic ability. Data represent mean ± S.D. from three independent experiments. **g** Transwell assay demonstrated that the metastatic ability of ccRCC cells was inhibited when rhCCL3 was added exogenously (30 ng/ml). Data represent mean ± S.D. from three independent experiments. **h** Wound healing assay illustrated that exogenously added rhCCL3 (30 mg/ml) promoted 786-O and OSRC-2 cells migration and invasion. Data represent mean ± S.D. from three independent experiments. **p < 0.01; ***p < 0.001.

CCL3 is the direct downstream target of piR-57125, so we firstly analyzed its expression in the TCGA sequencing data. The result confirmed that CCL3 was upregulated in ccRCC tissues compared to normal tissues (Fig. 4a) [25]. In addition, we found that CCL3 was highly expressed in ccRCC patients with axillary regional lymph node metastasis compared with those without lymphatic metastasis (Supplementary Fig. 6a) [26]. Our cohort also validated this finding (Fig. 4b and Supplementary Fig. 6b). Correlation analysis of piR-57125 levels and CCL3 mRNA levels in 21 pairs of ccRCC tissue samples identified a significant inverse correlation between piR-57125 and CCL3 (Fig. 4c). To determine whether CCL3 functions as an oncogene to promote ccRCC cell metastasis, we transfected 786-O and OSRC-2 cells with CCL3 siRNAs and verified the efficiency of CCL3 by western blot assay (Fig. 4d). The transwell assay showed that the metastasis ability of ccRCC was impaired by silencing of CCL3 expression (Fig. 4e). Similarly, wound healing assay also confirmed the same results (Fig. 4f). Meanwhile, we found that recombinant human CCL3 (rhCCL3, 30 ng/ml) promoted 786-O and OSRC-2 metastasis, as evaluated by the transwell assay and wound healing assay (Fig. 4g and h). In summary, these data indicated that CCL3 was overexpressed in ccRCC and promoted ccRCC cells metastasis.

### piR-57125 restrains ccRCC cell metastasis by inhibiting CCL3 expression

**Fig. 5:**
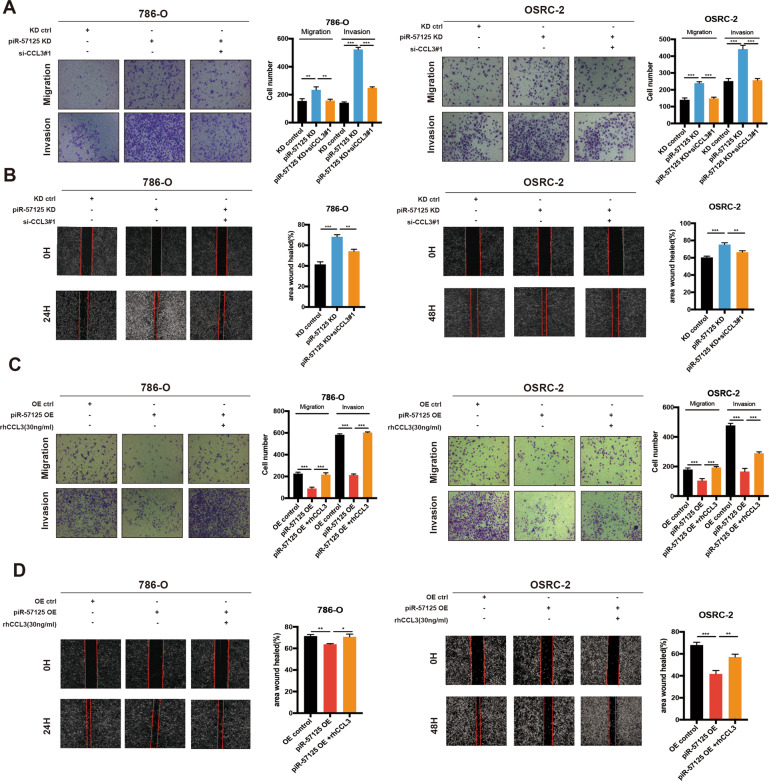
piR-57125 inhibits ccRCC metastasis through CCL3. **a**, **b** Transwell and wound healing assays revealed the effect of CCL3 knockdown on 786-O and OSRC-2 cells metastasis induced by knockdown of piR-57125. Data represent mean ± S.D. from three independent experiments. **c**, **d** Transwell and wound healing assays demonstrated the effect of overexpression of CCL3 on 786-O and OSRC-2 cells metastasis induced by overexpression of piR-57125. Data represent mean ± S.D. from three independent experiments. *p < 0.05; **p < 0.01; ***p < 0.001.

Since piR-57125 could directly bind to CCL3, we wondered whether CCL3 was involved in mediating the migration effect of piR-57125 in ccRCC cells. 786-O and OSRC-2 cells were transfected with piR-57125 inhibitors and CCL3 siRNA. Notably, the enhanced migration and invasion ability mediated by piR-57125 inhibitors was reversed, at least partially reversed, by co-transfection with CCL3 siRNA#1(Fig. 5a and b). A similar rescue effect was observed when transfection with piR-57125 mimics combined with recombinant human CCL3, the inhibitory effect of the piR-57125 mimics was abolished by the exogenous added rhCCL3 in ccRCC cells (Fig. 5c and d). Together, these results suggested that piR-57125 repressed ccRCC cells migration and invasion, at least partially, through inhibition of CCL3.

### piR-57125 functions as a tumor suppressor via downregulating the AKT/ERK signaling pathway

**Fig. 6:**
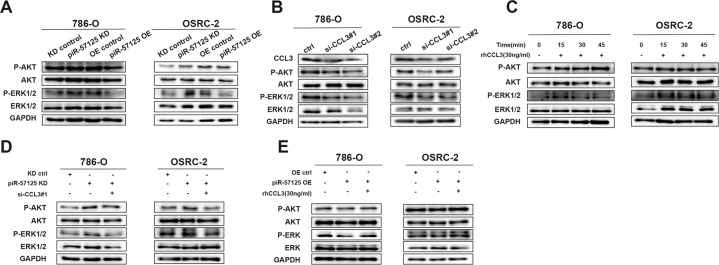
piR-57125 inhibits ccRCC metastasis via the AKT/ERK pathway. **a** Western blot assay showed the elevated or decreased expression of p-AKT and p-ERK1/2 upon knockdown or overexpression of piR-57125 in 786-O and OSRC-2 cells. **b**, **c** Western blot assay showed that the expression of p-AKT and p-ERK1/2 was decreased (**b**) or elevated (**c**) in ccRCC cells through transfected CCL3 siRNAs (**b**) or added rhCCL3 (**c**). **d**, **e** Western blot assay demonstrated that the increased **d** or decreased **e** expression of p-AKT and p-ERK1/2 induced by piR-57125 inhibitors **d** or mimics **e** could be reversed by CCL3 siRNAs (**d**) or rhCCL3 **e**.

Previous studies have elucidated that CCL3 regulates cell metastasis through the AKT/ERK pathway [27, 28]. We therefore hypothesized that the AKT/ERK pathway might be involved in piR-57125- and CCL3- induced ccRCC cell metastasis. Western blot analysis showed that the expression of AKT phosphorylation and ERK1/2 phosphorylation were elevated after treatment with piR-57125 inhibitors, while expression of AKT phosphorylation and ERK1/2 phosphorylation were downregulated after treatment with piR-57125 mimics (Fig. 6a). Similar results were observed in 786-O and OSRC-2 cells when transfected with CCL3 siRNA or treated with rhCCL3. Knockdown of CCL3 significantly decreased the levels of AKT phosphorylation and ERK1/2 phosphorylation and vice versa (Fig. 6b and c). Next, to determine whether CCL3 played an important role in the relationship between piR-57125 and the AKT/ERK pathway, we co-transfected ccRCC cells with piR-57125 inhibitors and CCL3 siRNAs. Strikingly, the promotion effect induced by piR-57125 inhibitors could be reversed by siCCL3 (Fig. 6d). We verified a similar rescue effect when cells were transfected with piR-57125 mimics combined with rhCCL3 (Fig. 6e). Collectively, these results revealed that piR-57125 regulated ccRCC cell metastasis through the CCL3/AKT/ERK signaling pathway.

### Knockdown of piR-57125 promotes ccRCC cells metastasis in vivo

**Fig. 7:**
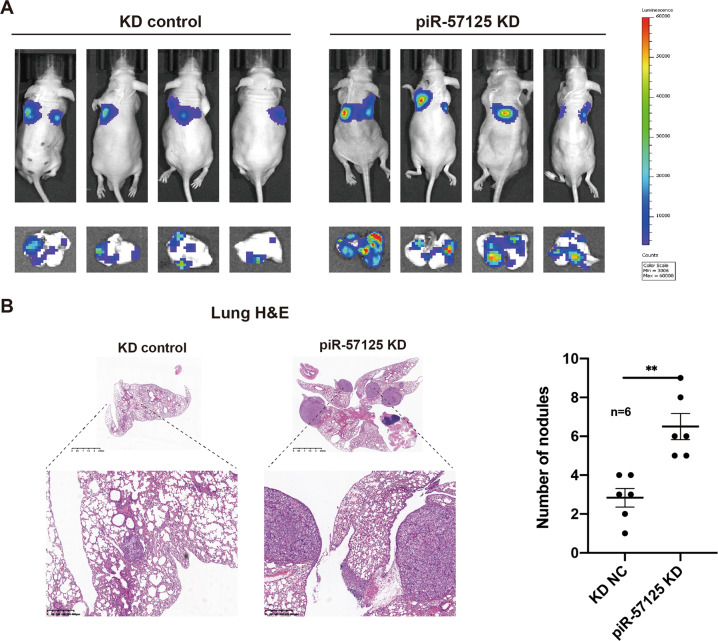
piR-57125 inhibits ccRCC metastasis in vivo. **a** Representative bioluminescence images showed that inhibition of piR-57125 promoted OSRC-2 cells lung metastasis in BALB/c nude mice 6 weeks after tail vein injection with the indicated cells. **b** H&E staining confirmed that inhibition of piR-57125 induced more lung metastatic lesions. Scales bars, 2.5 mm (top) and 400 um (bottom). Data represent mean ± S.D. **p < 0.01.

To investigate the effects of piR-57125 on ccRCC metastasis in vivo, we established a nude mouse lung metastasis model by tail vein injection of piR-57125 knockdown or scramble OSRC-2 cells. After 6 weeks of injection, the luminescence of lung metastasis foci was significantly enhanced in piR-57125 knockdown cells compared to scramble cells (Fig. 7a). We harvested the lungs of the nude mice and further evaluated the metastasis foci. We found that knockdown of piR-57125 formed more metastatic foci than were present in the scramble group (Supplementary Fig. 7a). Consistently, hematoxylin and eosin (H&E) staining also showed that knockdown piR-57125 significantly increased the number of pulmonary metastasis foci (Fig. 7b). Moreover, we found that piR-57125 was downregulated, while CCL3 was upregulated in the metastatic foci, which coordinated with the expectation (Supplementary Fig. 7b). Taken together, these results revealed that piR-57125 plays a tumor-suppressor role to inhibit ccRCC metastasis in vivo.

## Discussion

Recently, increasing evidence has shown that piRNAs play an important role in human diseases. Dysregulation of piRNAs is evidently a factor in the occurrence and development of cancers [11, 29]. In the current study, we identified piR-57125 as a metastasis-related piRNA; it is downregulated in the ccRCC tissues compared to the adjacent normal tissues. In vivo and in vitro assays have showed that knockdown of piR-57125 significantly promotes ccRCC metastasis, while overexpression of piR-57125 suppresses the metastasis ability of ccRCC. Thus, piR-57125 has a tumor-suppressor role in ccRCC metastasis and could be a metastatic-related biomarker that needed further validation.

Only a few previous studies have shown the association of piRNAs with ccRCC [14–16], and these articles are all focused on the diagnostic and prognostic significance of piRNAs in ccRCC. For example, Li et al. identified 3 piRNAs (piR-32051, piR-39894 and piR-43607) as being related to ccRCC metastasis, and therefore as potential diagnostic biomarkers for ccRCC [15]. In this study, we used the TCGA database along with piRNA sequencing to identify the functional piRNAs in ccRCC, and selected piR-57125 as the candidate piRNA. The functional assay showed that downregulation of piR-57125 promoted ccRCC metastasis both in vivo and in vitro, suggesting that piR-57125 could indeed be a functional piRNAs in ccRCC metastasis.

Although the research on piRNAs in tumorigenesis is still limited, the growing number of studies in recent years has revealed that piRNAs play an important role in cancer at the transcriptional and post-transcriptional levels [30, 31]. For example, Pi-sno75 has been reported to bind to the specific site of the target gene by increasing the H3K4me3 level and decreasing the H3K27me3 level to activate the expression of the gene [32]. Another study showed that piR-36712 was able to bind PIWIL1 to form piRISC, and subsequently degraded the SEPW1P RNA in a miRNA-like manner [32]. In addition, piRNAs/piwi complex could interact with certain proteins through piRNAs or PIWIL protein. piR-823 reportedly interacts with HSF1, promoting its phosphorylation at Ser326 and inducing HSF1 activation [33]. Since noncoding RNAs have been reported to exert their functions via cis-regulation, we first examined the effect of piR-57125 on genes nearby the piR-57125 gene locus. Unfortunately, we found that piR-57125 had little effect on their expression, suggesting that piR-57125 does not function in this way. Also, several studies have hinted that many piRNAs exerted their functions through DNA methylation; we therefore assessed the expression of DNMTs in the case of piR-57125 knockdown or overexpression. However, manipulating the expression of piR-57125 did not interfere with the expression of DNMTs. Then, we determined that piR-57125 was exist both in the cytoplasm and the nucleus, and the RIP assay revealed that piR-57125 could interact with PIWIL4, which suggested that piR-57125 could function in a miRNA-like manner. By RNA sequencing, we analyzed the downstream genes regulated by piR-57125, and identified CCL3 as a downregulated gene after piR-57125 overexpression. Surprisingly, bioinformatics analysis pointed out that piR-57125 could bind to the CCL3 mRNA at its 3’UTR, and the prediction was verified by dual-luciferase reporter gene assays. The qRT-PCR and western blot assay also showed that piR-57125 could decrease the mRNA and protein levels of CCL3. All these results demonstrate that piR-57125 can interact with PIWIL4 to form piRISC, resulting in the degradation of CCL3 mRNA.

CCL3, also known as macrophage inflammatory protein 1 alpha (MIP-1α), is an important chemokine that participated in immune surveillance and tolerance [34]. CCL3 has been reported to be upregulated in various types of cancers, including chondrosarcoma, non-small cell lung cancer, and oral squamous cell carcinoma, and functions as an oncogene [28, 35–37]. Zhang and colleagues found that upregulation of CCL3 promoted lung cancer metastasis [36]. In addition, a recent study showed that CCL3 promoted esophageal squamous cell migration and invasion through elevating the phosphorylation of AKT and ERK [28]. A similar effect was also observed in our study, as we found that CCL3 was upregulated in ccRCC compared to normal samples. Furthermore, knockdown of CCL3 significantly reduced the metastatic ability of ccRCC cells, and exogenous recombinant human CCL3 (rhCCL3) promoted ccRCC metastasis. Likewise, we found that inhibition of CCL3 could decrease ccRCC metastasis by reducing the phosphorylation level of AKT and ERK, as verified by WB assay. Rescue experiments revealed that inhibition of CCL3 could partially reverse the enhanced metastatic ability of ccRCC cells induced by piR-57125 knockdown, indicating that piR-57125 restrains ccRCC metastasis by inhibiting the expression of CCL3.

Although several dysregulated piRNAs have been reported in ccRCC previously, no study has explored the functional roles of piRNAs in ccRCC. This is the first study to investigate the function of piR-57125 in ccRCC. Also, the piR-57125/CCL3/AKT/ERK pathway explains the specific reasons why piR-57125 restrains ccRCC progression. However, there are still some limitations to this study. For example, although many researchers have reported that CCL3 participates in the AKT/ERK pathway, we still do not know the specific mechanism by which CCL3 regulates the AKT/ERK pathway, and more attention should be focused on this area.

In summary, our current research shows that piR-57125 is downregulated in the ccRCC tissues. Functionally, piR-57125 can inhibit ccRCC metastasis both in vivo and in vitro. Mechanistically, piR-57125 suppresses ccRCC metastasis through downregulation of the CCL3/AKT/ERK axis. Overall, piR-57125 is a potential therapeutic target for the diagnosis and treatment of metastatic ccRCC.

## Materials and Methods

### piRNA sequencing

Two paired ccRCC and adjacent normal tissue samples were obtained from surgical specimens at the Department of Urology, Sir Run Run Shaw Hospital. Sample preparation was performed according to the instruction of Illumina NextSeq500’s. The total RNA of each sample was sequentially ligated to 3’ and 5’ small RNA adapters. After amplification, ~142–153 bp fragments were extracted and purified from the PAGE gel. Subsequently, the libraries were qualified using BioAnalyzer2100 (Agilent). Then, the libraries were denatured as single-strand DNA molecules, amplified in situ, and finally sequenced on Illumina NextSeq following the manufacturer’s instructions.

### Tissue samples

We obtained 45 paired clear-cell renal cell carcinoma samples and adjacent normal tissue samples at Sir Run Run Shaw Hospital, School of Medicine, Zhejiang University between 2013 and 2019. None of the patients had received chemotherapy or radiotherapy before surgery. This study was approved by the Ethics Committee of Sir Run Run Shaw Hospital, School of Medicine, Zhejiang University. The clinical characteristics of all patients are summarized in Supplementary Table S2 and informed consent was obtained.

### Cell culture

Both ccRCC cell lines, 786-O and OSRC-2, were purchased from the Institute of Biochemistry and Cell Biology of the Chinese Academy of Sciences (Shanghai, China). 786-O and OSRC-2 are cultured in RPMI-1640 supplemented with 10% fetal calf serum (Invitrogen) in humidified air at 37 °C, with 5% CO_2_.

### Cell transfection

RCC cell lines were transfected with piRNA inhibitors, mimics, and siRNA using RNAimax (Invitrogen), and transfected with plasmids using Lipofectamine 3000 (Invitrogen). The piR-57125 inhibitors, mimics were designed and synthesized by RiboBio (Guangzhou, China). And the siRNAs targeting CCL3 were chemically synthesized by RiboBio (Guangzhou, China), the corresponding sequence was: siCCL3#1: 5’-CCGGCAGATTCCACAGAAT-3’; siCCL3#2: 5’-CAACCAGTTCTCTGCATCA-3’. The Recombinant Human CCL3 protein was purchased from R&D systems (270-LD, R&D systems). 48 h after post-transfection, cells were harvested for Western blot analysis or qRT-PCR.

### RNA extraction and qRT-PCR analysis

Total RNA of cells and tissues was extracted with TRIzol reagent (CWBio), following the manufacturer’s instructions. The miRNA 1st strand cDNA synthesis kit (MR101, Vazyme) was used to amplify the specific piRNA with piRNA stem-loop primers. Normal cDNA was synthesized using the HiFiScript cDNA Synthesis Kit (CWBio). mRNA and piRNA expression levels were determined by qRT-PCR on a Roche Light Cycler 480 instrument using the SYBR Green method. The mRNA levels were normalized to the expression of GAPDH, and the piR-57125 levels were normalized to U6 small RNA. The qPCR results were analyzed with the 2−ΔΔCt method. All the primer sequences are listed in Supplementary Table S3.

### Cytoplasmic and nuclear fractionation

Cytoplasmic and nuclear RNAs of 786-O and OSRC-2 cells were isolated and purified using the Cytoplasmic & Nuclear RNA Purification Kit (Norgen Biotek).

### Western blot and antibody

Protein exacts from cells were prepared using RIPA extraction reagent, and proteins (50ug) were separated by 8–12% SDS-PAGE and followed transferred onto PVDF membranes (Millipore, Billerica, MA), and probed with specific primary antibodies overnight at 4 °C. The specific antibody information is listed in Supplementary Table S3. Finally, the bands were visualized using the ECL chemiluminescent detection system (Thermo Fisher Scientific).

### Transwell assay

The cell migration and invasion assays have been described previously [38, 39]. Briefly, 2 × 10^4^ ccRCC cells were seeded on the upper chambers coated with or without 50 μL Matrigel in the serum-free medium after 48 h transfected with piR-57125 inhibitors or mimics, while RPMI 1640 medium with 10% FBS was added in the lower chambers. After twenty-four hours, cells in the lower membrane were fixed and stained with crystal violet (0.1%), then visualized under a microscope at 100x magnification. This assay was repeated with 3 times.

### Wound healing assay

786-O and OSRC-2 cells were seeded in the 6-well plates. Upon reaching 100% confluence as a monolayer, a wound was made by using 200 ul pipette tip on the cell monolayer and photographed as 0 h. Subsequently, the cells were cultured in the fresh medium without serum and photographed at the appropriate time to calculate the occupied area by migrated cells.

### RNA sequencing

Total RNA was extracted with TRIzol reagent (CWBio) following the manufacturer’s instructions. The purity of RNA was assessed using the ND-1000 NanoDrop, and the RNA integrity was assessed using the Bioanalyzer 2100 system (Agilent Technologies, CA, USA). Briefly, fragmentation was carried out using divalent cations in First-Strand Synthesis Buffer (5X). First-strand and second-strand cDNA synthesis were performed according to the manufacturer’s instructions, followed by adaptor ligation. Then the library fragments were purified with the AMPure XP system (Beckman Coulter, Beverly, USA). PCR was performed, and the PCR products were purified (AMPure XP system) and assessed on the Bioanalyzer 2100 system. The clustering of samples was performed using the TruSeq PE Cluster Kit v3-cBot-HS (Illumia) according to the manufacturer’s instructions. The library preparations were sequenced on an Illumina Novaseq platform and 150 bp paired-end reads were generated. After removing reads containing the adapter, the clean data were obtained. Then Hisat2 v2.0.5 was used as a mapping tool to aligned the clean reads to the reference genome. FeatureCounts v1.5.0-p3 was used to count the read numbers mapped to each gene. Differential expression analysis was performed using the DESeq2 R package (1.20.0). Genes with an adjusted *P*-value < 0.05 were assigned as differentially expressed.

### ELISA

786-O and OSRC-2 cells were seeded in the 6-well plates. After 48 h transfected with piR-57125 mimics or inhibitors. The cells were then washed with PBS and cultured in fresh serum-free media until 80% of confluency. Supernatants were harvested 24 hr later and used for the subsequent ELISA assay. The CCL3 ELISA kit was purchased from RayBiotech, and the experiments were performed according to the manufacturer’s instructions.

### Animal model and in vivo imaging

To test the metastatic effect of piR-57125, BALB/c nude mice were used for the experiment. A total of 5 × 10^6^ luciferase-labeled OSRC-2 cells with piR-57125 knockdown were injected via the tail vein to each 4–5 weeks old mice (*n* = 6). Tumor development and metastasis were measured through in vivo imaging system (IVIS) after 6–8 weeks. At that point, the mice were euthanized, and the metastases loci were removed and subjected to H&E staining. All experimental procedures were performed in accordance with relevant institutional and national guidelines and regulations.

### RNA immunoprecipitation (RIP) assay

RNA immunoprecipitation assay was performed using the MagnaRIP RNA RIP RNA-Binding Protein Immunoprecipitation Kit (Millipore) according to the manufacturer’s instructions. The total immunoprecipitation RNA was detected by qRT-PCR.

### Luciferase reporter assay

We constructed a luciferase reporter vector containing wild-type or mutant 3’UTR of CCL3 were constructed. 293 T cells were grown in a 24-well plate and transfected with luciferase vector and piR-57125 mimics using Lipofectamine 3000 (Invitrogen) or RNAimax (Invitrogen) transfection reagent, respectively. After 48 h of transfection, the cells were collected and measured, using the Dual-Luciferase Reporter Assay System (Promega) according to the manufacturer’s instructions. Renilla luciferase activity was normalized to firefly luciferase activity. Transfection was repeated in triplicate.

### Statistical analysis

The results were reported by the means ± SDs. Statistical analyses were performed using GraphPad Prism 7 (GraphPad Software, Inc., CA). The significance of the difference between the groups was calculated by the Student’s *t* test and one-way analysis of variance (ANOVA) test, and *P* < 0.05 was considered statistically significant.

## Supplementary information

Supplementary Fig

Supplementary Table S1 ALL_Differentially_Expressed_piRNA

Supplementary Table S2 patients clinical information

Supplementary Table S3 primer and antibody information

## Data Availability

The datasets used and/or analyzed during the current study are available from the corresponding author on reasonable request.
