# Supplementary material for: PIWI-interacting RNA 57125 restrains clear cell renal cell carcinoma metastasis by downregulating CCL3 expression
Source: Cell Death Discov. 2021 Nov 3;7:333. doi: 10.1038/s41420-021-00725-4 (PMC8566597; doi:10.1038/s41420-021-00725-4)
Supplement: Supplementary file 1 — Supplementary Fig [file 41420_2021_725_MOESM1_ESM.docx]

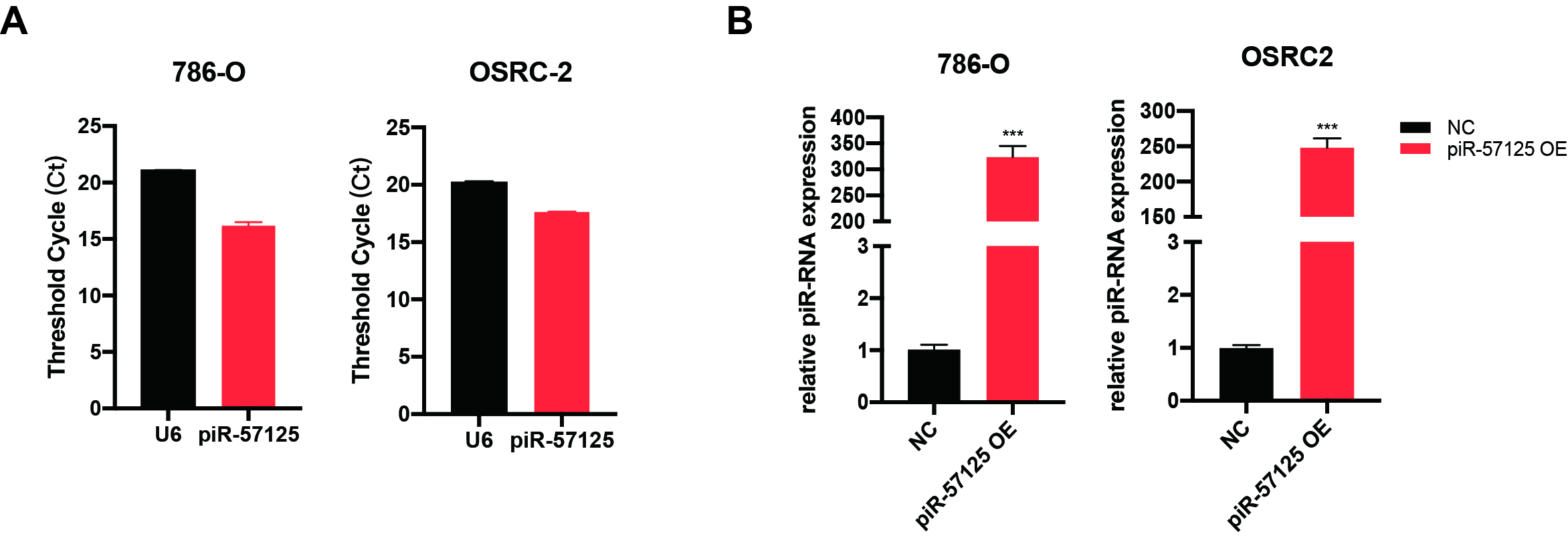


**Supplementary Fig.1** Expression of piR-57125 in 786-O and OSRC-2 cell lines, and verification of piR-57125 overexpression efficiency. **a,** relative expression of piR-57125 in compared with U6. **b,** qRT-PCR assay shows the overexpression efficiency of piR-57125 in 786-O and OSRC-2 cells. Data represent mean±S.D. from three independent experiments. ***, p<0.001.


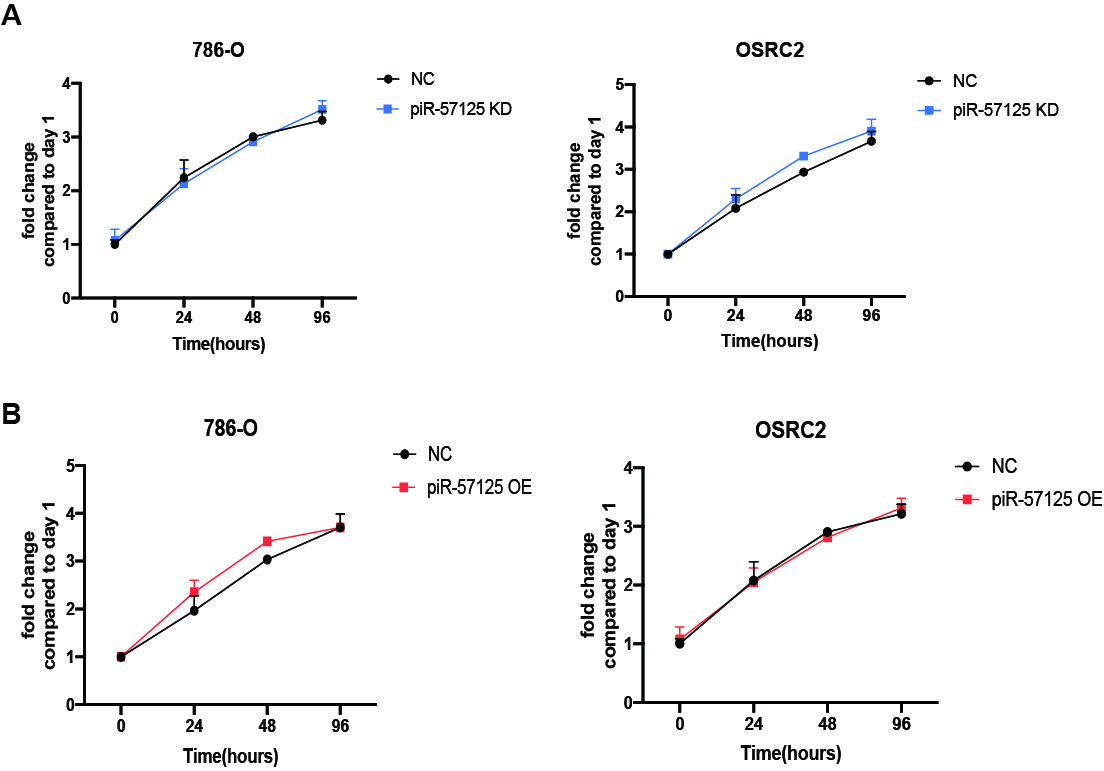


**Supplementary Fig.2** piR-57125 do not alter the proliferation rate of ccRCC. **a, b,** CCK-8 assay shows knockdown **(a)** or overexpression **(b)** of piR-57125 have little effects on 786-O and OSRC-2 cells proliferation. Data represent mean±S.D. from three independent experiments.

**
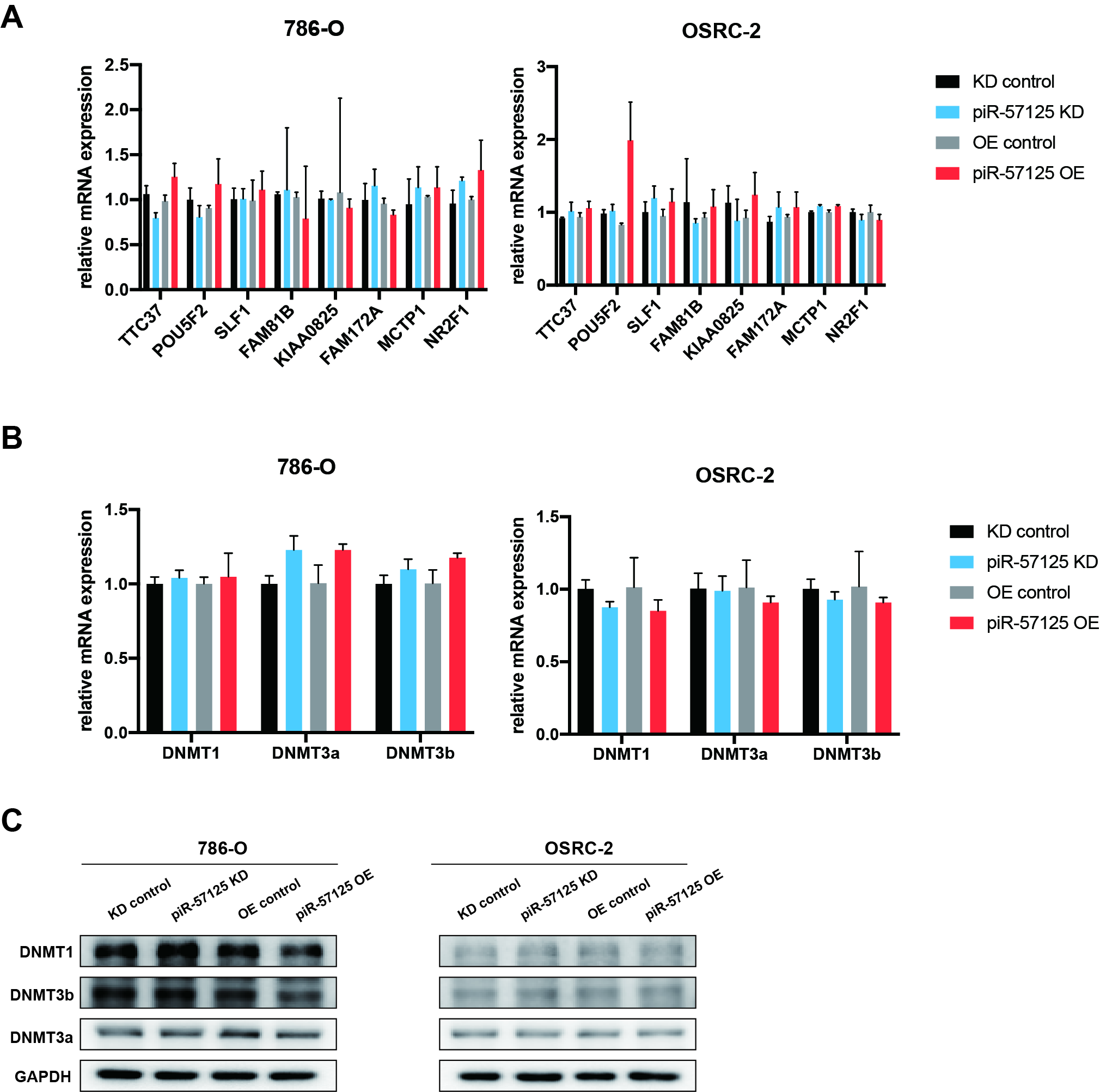
**

**Supplementary Fig.3 a**, qRT-PCR assay demonstrates that the expression of neighboring genes near piR-57125 genome locus did not altered after knockdown or overexpression of piR-57125. Data represent mean±S.D. from three independent experiments. **b, c,** qRT-PCR **(b)** and western blot **(c)** assays reveal that knockdown or overexpression of piR-57125 do not change the expression of DNMTs. Data represent mean±S.D. from three independent experiments.


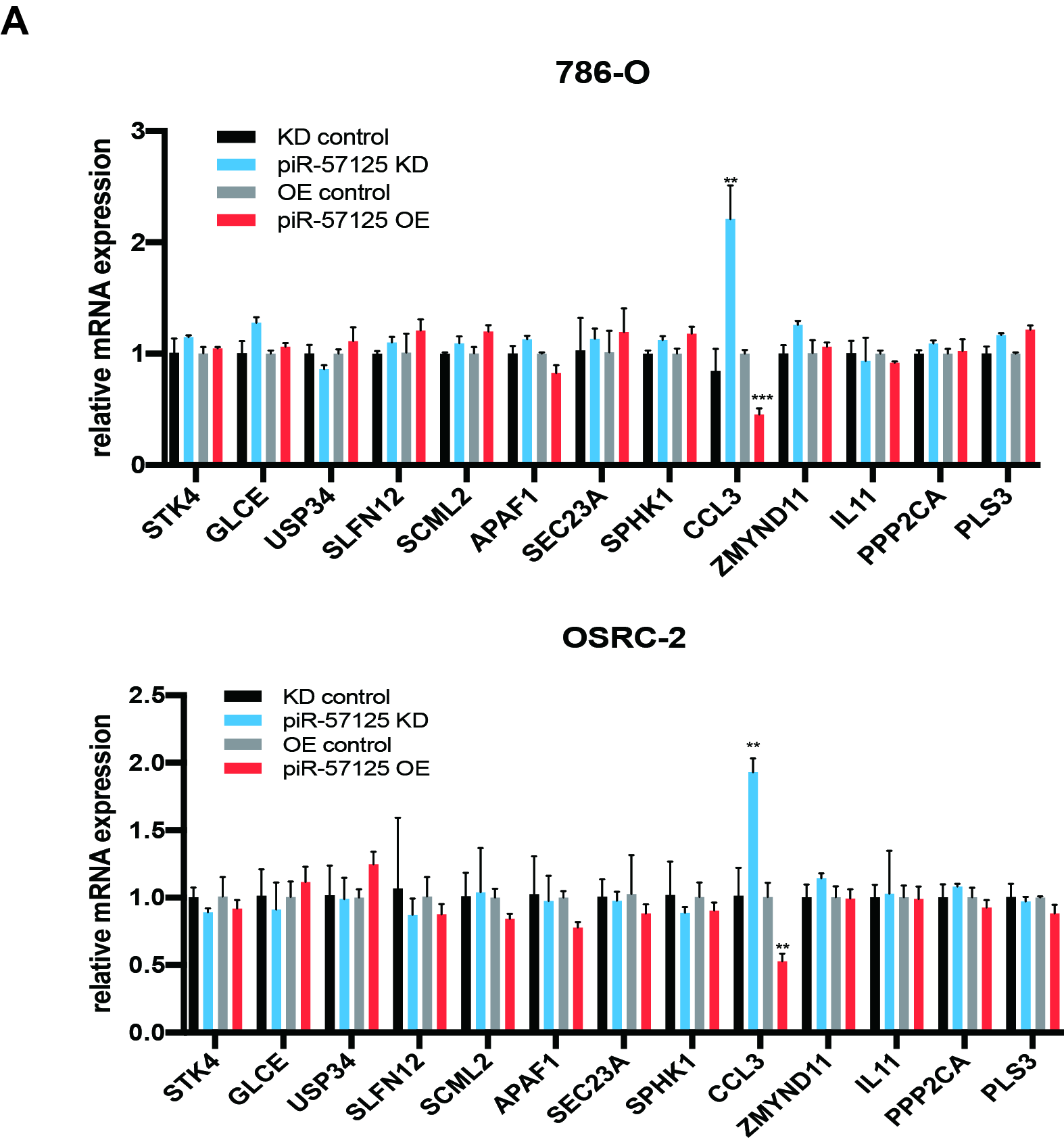


**Supplementary Fig.4 a,** The potential targets of piR-57125 selected from RNA-seq were verified by qRT-PCR, and the results show only CCL3 meet the result of RNA-seq. Data represent mean±S.D. from three independent experiments. **, p<0.01; ***, p<0.001.


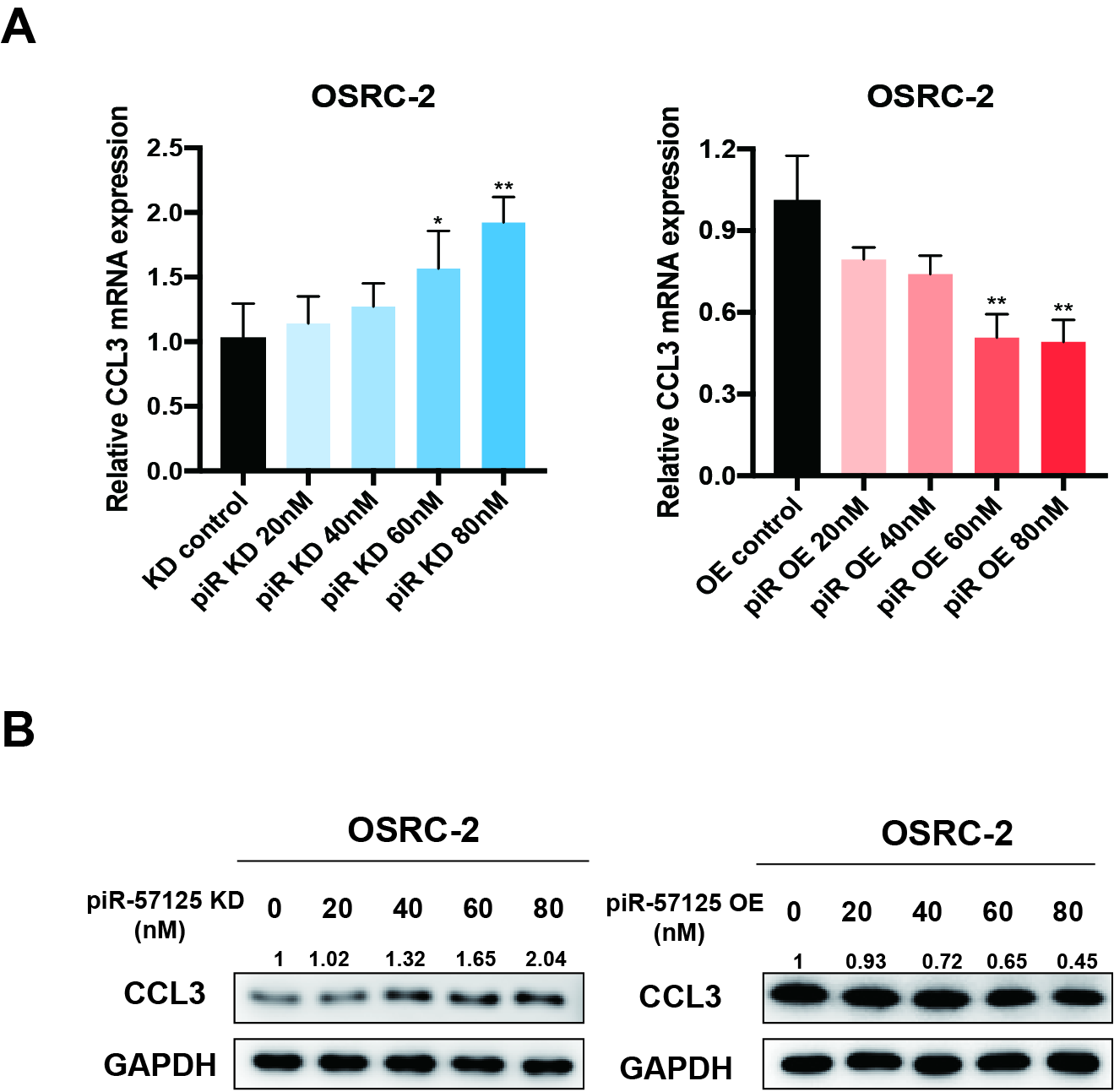


**Supplementary Fig.5 a,b** Decreased or increased mRNA (**a**) or protein (**b**) expression of CCL3 in OSRC-2 cells via transfection with piR-57125 inhibitors or mimics respectively. Data represent mean±S.D. from three independent experiments.


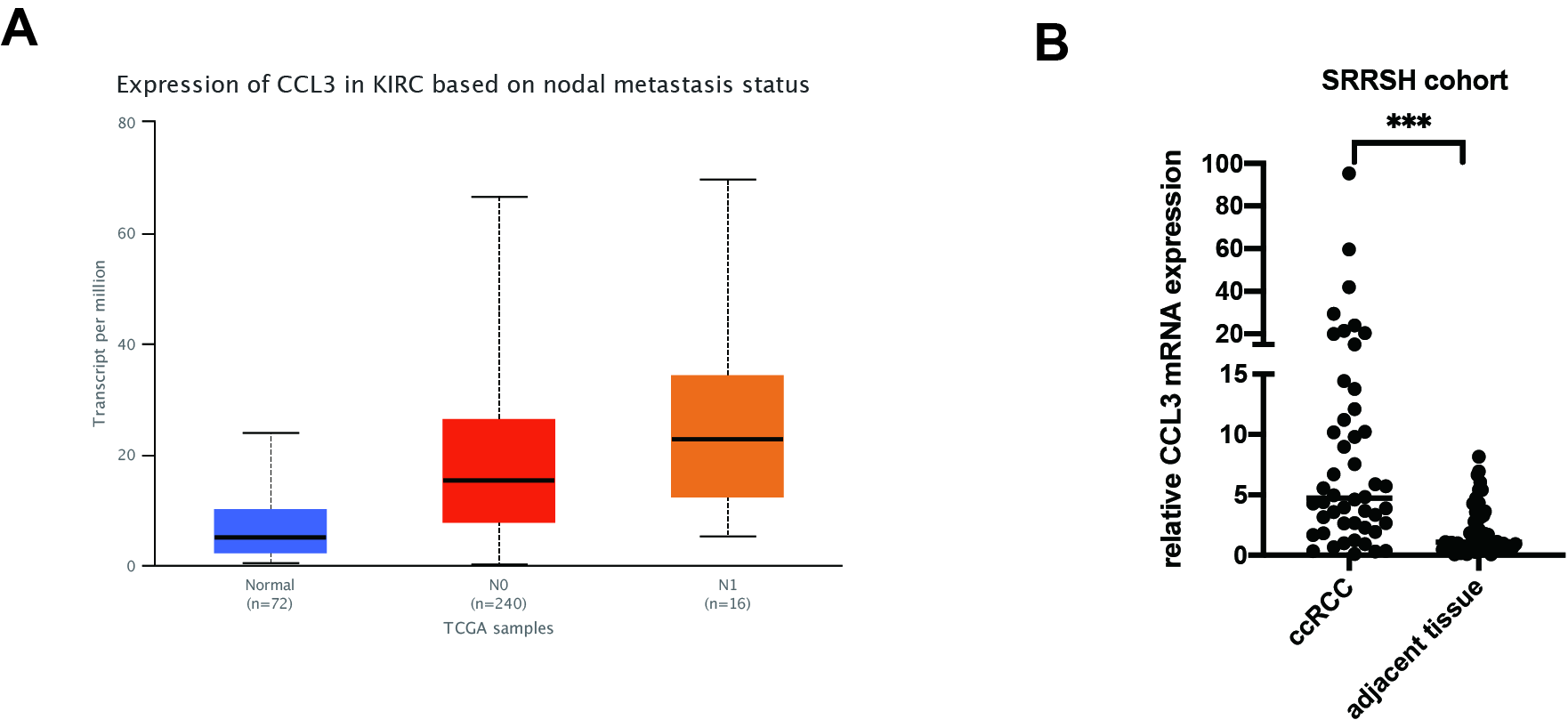


**Supplementary Fig.6 a,** the expression of CCL3 in ccRCC based on nodal metastasis status was analyzed based on TCGA data. **b,** Dot blot shows the expression of CCL3 in 45 paired ccRCC and adjacent normal samples from patients recruited from the SRRSH cohort. ***, p<0.001.

**
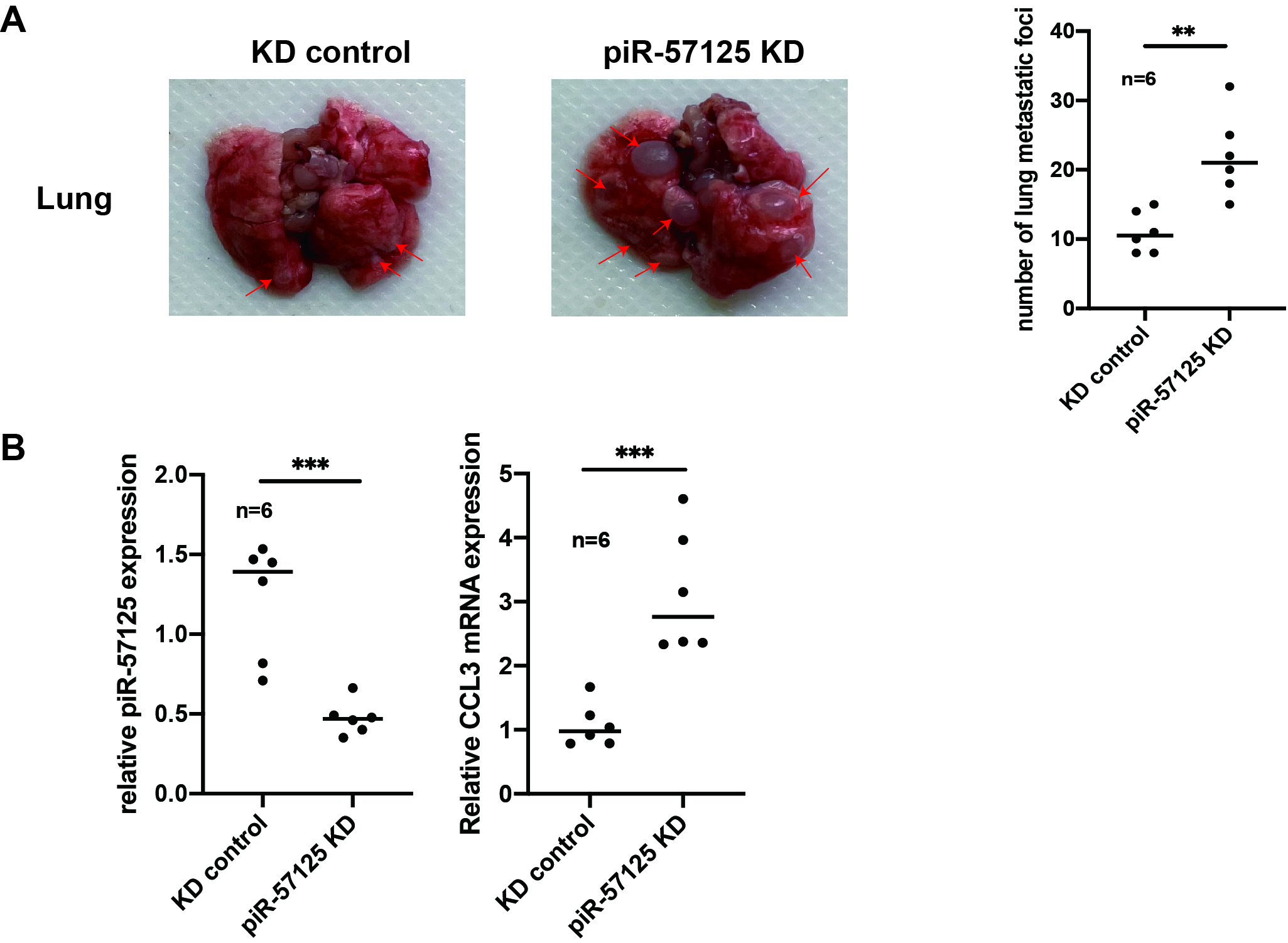
**

**Supplementary Fig.7 a,** After 6 weeks injection of piR-57125 KD cells or control cells through tail vein, the lung specimens were collected, the red arrows indicate metastatic foci. **b,** qRT-PCR assays show that piR-57125 is downregulated, while CCL3 is upregulated in lung metastatic foci in piR-57125 KD group. **, p<0.01; ***, p<0.001.
