# Supplementary material for: PIWI-interacting RNA 57125 restrains clear cell renal cell carcinoma metastasis by downregulating CCL3 expression
Source: Cell Death Discov. 2021 Nov 3;7:333. doi: 10.1038/s41420-021-00725-4 (PMC8566597; doi:10.1038/s41420-021-00725-4)
Supplement: Supplementary file 4 — Supplementary Table S3 primer and antibody information [file 41420_2021_725_MOESM4_ESM.docx]

Primers used for quantitative Real Time-PCR in this study.

| Gene Symbol | Forward primer (5’→3’) | Reverse primer (5’→3’) | |
| --- | --- | --- | --- |
| *FAM172A* | *ACCGCCTCTTGATTTTCCTGA* | *GCCTCGTATCTTTTCTGGTTCC* | |
| *NR2F1* | *ATCGTGCTGTTCACGTCAGAC* | *TGGCTCCTCACGTACTCCTC* | |
| *POU5F2* | *GACATCTCGGGCATACTGAA* | *TCGTCTGGCTAAGCACCTT* | |
| *KIAA0825* | *AAGCTGGGTCCTTGGTTC* | *GGACTGAAAGCCGAGGGT* | |
| *MCTP1* | *GGAAAGATAACAGGCAACG* | *ACCTCCTGGATGGCATAG* | |
| *SLF1* | *CAAGTTGCTGGTAATGAAAA* | *ACAGAGGAATGCTAAGGGAG* | |
| *FAM81B* | *GGATTATCGCCACGAAATGAACC* | *GTTTCTCCTGTAGGACCTTCAGA* | |
| *TTC37* | *GGAAGGTGTTATGGTCGTG* | *TGCTGCCTGTTTCTGCTA* | |
| *DNMT1* | *AGGCGGCTCAAAGATTTGGAA* | *GCAGAAATTCGTGCAAGAGATTC* | |
| *DNMT3a* | *CCGATGCTGGGGACAAGAAT* | *CCCGTCATCCACCAAGACAC* | |
| *DNMT3b* | *AGGGAAGACTCGATCCTCGTC* | *GTGTGTAGCTTAGCAGACTGG* | |
| *PLS3* | *TGGCTACCACTCAGATTTCCA* | *TCACAAATGAATCCGTTGCTGT* | |
| *CCL3* | *AGTTCTCTGCATCACTTGCTG* | *CGGCTTCGCTTGGTTAGGAA* | |
| *PPP2CA* | *CAAAAGAATCCAACGTGCAAGAG* | *CGTTCACGGTAACGAACCTT* | |
| *SEC23A* | *GGAGTCCGATTTAGTTGGAATGT* | *AGGTCTCTCTTTCAGTGGTGT* | |
| *SPHK1* | *GCTCTGGTGGTCATGTCTGG* | *CACAGCAATAGCGTGCAGT* | |
| *ZMYND11* | *ATGGCACGTTTAACAAAAAGACG* | *CGGTCAATGTTGGCAATCTGC* | |
| *IL11* | *ACATGAACTGTGTTTGCCGC* | *AGCTGGGAATTTGTCCCTCAG* | |
| *STK4* | *CCTCCCACATTCCGAAAACCA* | *GCACTCCTGACAAATGGGTG* | |
| *GLCE* | *GCAGCTCGGGTCAACTATAAG* | *GAACGCCGTGGAAACTGGA* | |
| *USP34* | *TGGCTACATATTCCCGCTGTC* | *GCTGCACTCTGTCGTAACTCC* | |
| *SLFN12* | *TTGGAAACGAATTATGCCGAGT* | *AGAGCACACATAGCTCGTGAG* | |
| *SCML2* | *AGGGATGATTTCCACTGGGAG* | *ACTGACGGAAGCACTCTGAAG* | |
| *APAF1* | *AAGGTGGAGTACCACAGAGG* | *TCCATGTATGGTGACCCATCC* | |
| *GAPDH* | *CAAGGTCATCCATGACAACTTTG* | *GTCCACCACCCTGTTGCTGTAG* | |
| Primers used for stem-loop RT-PCR of piRNAs | | | |
| Gene Symbol | Stem-loop Reverse transcription primers (5’→3’) | Forward primer (5’→3’) | Reverse primer (5’→3’) |
| *piR-57125* | *GTCGTATCCAGTGCGTGTCGTGGAGTCGGCAATTGCACTGGATACGACTTCTCGCA* | *TGGTCGTGGTTGTAGTCCG* | *CAGTGCGTGTCGTGGAGT* |
| *U6* | *AACGCTTCACGAATTTGCGT* | *CTCGCTTCGGCAGCACA* | *AACGCTTCACGAATTTGCGT* |

Antibody used in this study

| Antibody | company |
| --- | --- |
| *GAPDH* | *abcam* |
| *PIWIL4* | *abcam* |
| *PIWIL1* | *abcam* |
| *PIWIL2* | *abcam* |
| *CCL3* | *abcam* |
| *ERK1/2* | *abcam* |
| *phospho-ERK1/2* | *abcam* |
| *DNMT1* | *abcam* |
| *DNMT3a* | *abcam* |
| *DNMT3b* | *abcam* |
| *AKT* | *CST* |
| *phospho-AKT* | *CST* |
